# ST3GalIV drives SLeX biosynthesis in gastrointestinal cancer cells and associates with cancer cell motility

**DOI:** 10.1007/s10719-023-10113-y

**Published:** 2023-04-19

**Authors:** Ana F. Costa, Emanuel Senra, Isabel Faria-Ramos, Andreia Teixeira, João Morais, Mariana Pacheco, Celso A. Reis, Catarina Gomes

**Affiliations:** 1 I3S–Instituto de Investigação e Inovação em Saúde, University of Porto, Porto, Portugal; 2 IPATIMUP–Institute of Molecular Pathology and Immunology, University of Porto, Porto, Portugal; 3 Present Address: ICBAS-School of Medicine and Biomedical Sciences, University of Porto, Porto, Portugal; 4 Faculty of Science, University of Porto, Porto, Portugal; 5 Present Address: Faculty of Medicine, University of Porto, Porto, Portugal

**Keywords:** ST3GalIV, SLeX, Cancer cells, Cell motility

## Abstract

Expression of sialyl Lewis X (SLeX) is a well-documented event during malignant transformation of cancer cells, and largely associates with their invasive and metastatic properties. Glycoproteins and glycolipids are the main carriers of SLeX, whose biosynthesis is known to be performed by different glycosyltransferases, namely by the family of β-galactoside-α2,3-sialyltransferases (ST3Gals). In this study, we sought to elucidate the role of ST3GalIV in the biosynthesis of SLeX and in malignant properties of gastrointestinal (GI) cancer cells. By immunofluorescent screening, we selected SLeX-positive GI cancer cell lines and silenced ST3GalIV expression via CRISPR/Cas9. Flow cytometry, immunofluorescence and western blot analysis showed that ST3GalIV KO efficiently impaired SLeX expression in most cancer cell lines, with the exception of the colon cancer cell line LS174T. The impact of ST3GalIV KO in the biosynthesis of SLeX isomer SLeA and non sialylated Lewis X and A were also evaluated and overall, ST3GalIV KO led to a decreased expression of SLeA and an increased expression in both LeX and LeA. In addition, the abrogation of SLeX on GI cancer cells led to a reduction in cell motility. Furthermore, ST3GalVI KO was performed in LS174T ST3GalIV KO cells, resulting in the complete abolishment of SLeX expression and consequent reduced motility capacity of those cells. Overall, these findings portray ST3GalIV as the main, but not the only, enzyme driving the biosynthesis of SLeX in GI cancer cells, with a functional impact on cancer cell motility.

**Supplementary Information:**

The online version contains supplementary material available at 10.1007/s10719-023-10113-y.

## Introduction

Glycosylation is a major process that regulates several important features of all cells and plays a pivotal role in both health and disease [1, 2]. Through to the highly compartmentalized and coordinated action of hundreds of enzymes, a myriad of glycan structures is synthesized and can be found attached to distinct molecules such as proteins and lipids [3]. Sialic acids, usually present at the terminal end of glycan structures, play crucial roles in cellular adhesion [4] and communication [5], antibody-dependent cellular cytotoxicity [6, 7], and cancer development and progression [8].

It is well established that cancer cells express aberrant profiles of cellular glycans [9]. For instance, increased expression of sialylated glycans, such as the sialylated Lewis antigens SLeA and SLeX, has been vastly documented in the majority of cancer types. Usually, the increased expression of these glycans is associated with enhanced cancer cell motility and invasion [9–11], and further translates, *in vivo*, to patient’s poor survival [12]. In fact, the expression level of SLeA is routinely assessed trough the CA19.9 serological test to infer cancer presence, recurrence, and treatment follow-up.

The increased expression of SLeA and SLeX in cancer is mainly due to the expression of enzymes involved in their intracellular biosynthesis, namely the sialyltransferases (STs). STs are a group of glycosyltransferases that catalyze the addition of sialic acids to the terminal non-reducing ends of glycan structures. This family comprises several enzymes with distinct specificities that were already comprehensively described by Harduin–Lepers *et al*. [13]. SLeX, Neu5Acα2-3Galβ1-4[Fucα1-3]GlcNAc-R, is a terminal tetrasaccharide characterized by the addition of sialic acids in a α2,3 linkage to the galactose residue in type II structures. This addition is mainly performed by the members of the α2,3 ST subfamily, ST3Gal III, IV and VI [14].

SLeX is normally expressed in immune and inflammatory cells upon cytokine stimuli, triggered during inflammation, and is recognized as a selectin binding/ligand to endothelial cells. This sweet harmony leads to immune cell adhesion to endothelia and further facilitates cell rolling and extravasation to the inflammatory site [15]. In such a way, cancer cells that express SLeX take advantage of this process to easily travel through the bloodstream and form secondary metastasis in distant organ sites [11].

Previously, using a ST3GalIV overexpression cellular model, we reported the role of ST3GalIV in SLeX expression in gastric cancer cells [16, 17] and its association with the activation of c-Met signaling pathway [18]. More recently, we showed that SLeX is expressed in aggressive gastric carcinomas, highlighting its clinical value as a prognostic biomarker and an appealing therapeutic target [12]. These results compelled us to deeply explore the role of SLeX in cancer. In this study, we further elucidate the role of ST3GalIV in SLeX expression and the malignant properties of GI cancer cells, through the establishment of glycoengineered cellular KO models to comprehensively understand the specific contribution of distinct ST3Gals.

## Materials and methods

### Cell lines and cell culture

KatoIII, NCI-N87, SNU1, SNU638, Colo205, LS174T, HT-29, HCT15, HCT116, RKO, DLD1, SW48, SW480, SW620 and Caco-2 were obtained from American Type Culture Collection (ATCC). The cells were grown in monolayer culture in 6-well plates (Fisher Scientific) and maintained at 37 °C in an atmosphere of 5% CO_2_, in Roswell Park Memorial Institute (RPMI) 1640 GlutaMAX, HEPES medium, Dulbecco's Modified Eagle Medium (DMEM) or Iscove Modified Dulbecco Media (IMDM) (all from Gibco), all supplemented with 10% fetal bovine serum (FBS) (Biowest).

### RNA extraction and reverse transcription PCR

Total RNA was extracted from cellular pellets using the TRI Reagent (Sigma) protocol, according to manufacturer instructions. Isolated RNA was then quantified on a NanoDrop ND-1000 spectrophotometer (NanoDrop Technologies, Inc.) and converted to cDNA by reverse transcription (RT). RT was performed using a mixture containing 3 μg of total RNA, 1 μL of random oligonucleotides primers, 1 μL of 10 mM deoxynucleotide triphosphates (dNTPs), 4 μL of 5X RT buffer, 1 μL of 100 mM dithiothreitol (DTT), 1 μL of RNAseOUT (40 U/μL), 1 μL of SuperScriptR IV reverse transcriptase (200 U/μL) (Invitrogen) and nuclease-free water in a final reaction volume of 20 μL, according to the manufacturer's protocol. Briefly, RT reactions involve three main steps: primer annealing (65 °C, 5 min; 4 °C, > 1 min), DNA polymerization (23 °C, 10 min; 50–55 °C, 20 min) and enzyme deactivation (80 °C, 10 min). A mixture without RNA was used as control for RT.

### Real-time PCR (q-PCR) analysis

For real-time PCR analysis, 2 μL of cDNA samples (diluted 30-fold with Nuclease-Free Water), 0.6 μL of each 10 mM primer and 10.0 μL Power SYBRGreen Master Mix (Applied Biosystems) were amplified using an ABI Prism 7000 Sequence Detection System (Applied Biosystems). The primers used are summarized in Table 1. Expression analysis of ß-Actin housekeeping gene was also measured in triplicate for each sample and used for normalization of target gene abundance. Specificity of amplification was confirmed by melting curve analysis. Standard curves were determined for each gene, and results are presented as ratio between target gene and the housekeeping gene, ß-Actin.

**Table 1:** Primer sequences for qRT-PCR analysis of gene expression

*Gene*	Primer Foward	Primer Reverse
*ST3GAL3*	GGTGGCAGTCGCAGGATTT	CATGCGAACGGTCTCATAGTAGTG
*ST3GAL4*	TGAGGTGGCCCGAGG	CCGGGAGTAGTTGCCAAA
*ST3GAL*6	CGGCTGATTTTAGAAAGATTGCTT	CGGCTGATTTTAGAAAGATTGCTT
*ß-Actin*	AGAAAATCTGGCACCACACC	TAGCACAGCCTGGATAGCAA

### CRISPR/Cas9 knockout

*ST3GAL4 and ST3GAL6* knockout were performed using CRISPR/Cas9 technology as described previously [19]. Briefly, cells were co-transfected with a Cas9 endonuclease vector containing GFP and a plasmid with a validated guide RNA for the target genes (*ST3GAL4* “TTACCCGCTTCTTATCACTC” and *ST3GAL6* “TAATACGACAGTGATTCTCA”). KO clones were obtained by fluorescence-activated single-cell sorting (FACS) for the enrichment of nuclease-expressing cells, and gene KO was validated by indel detection using amplicon and restriction fragment length polymorphism (RFLP) combination analysis, as previously described [20]. Three clones were selected for each target gene and gene KO was confirmed by Sanger sequencing. The results were analyzed through Tracking of Indels by Decomposition (TIDE) methodology [21].

### Immunofluorescence

All cell lines and respective KO cell models were seeded in 12-well chamber slides (ibidi) or in 13 mm coverslips (Marienfeld). The cells were washed with phosphate-buffered saline (PBS) and fixed with methanol during 20 min at -20 °C. Blocking of immunoglobulins cross-reaction was performed using goat serum (1:5) (Dako*)* in 10% BSA in PBS at RT for 30 min, followed by primary antibody incubation at 4 °C overnight. Slides were then incubated with fluorescently-labeled secondary antibodies anti-mouse IgM Alexa Fluor® 594 (1:500) or IgG Alexa Fluor® 488 (1:750) (Thermo Fisher Scientific) for 1 h at RT. Nuclear counterstaining was performed by incubating cells with 4`,6`-diamino-2fenil-indol (DAPI) for 10 min and slides or coverslips were mounted with VectaShield (Vector Laboratories). The fluorescent signal was examined using a fluorescence microscope and images were acquired with Zeiss Axio Imager Z1, Zeiss AxioCam MR version 3.0, and the AxioVisionRel (version 4.8) software (Carl Zeiss). Images were acquired under 200x and 400x (Fig. 5 and S3) magnifications.

The primary antibodies used were: SLeX (CSLEX, 1:250, BD Bioscience,), SLeA (CA19-9, 1:200, Abcam,), LeX (SH1, 1:5, [22]) and LeA (CA3F4 1:5, [23]).

### Flow cytometry analysis

Cells previously cultured in 25cm2 flasks were detached with accutase (Gibco), resuspended in fresh medium and centrifuged at 300 g for 5 min. Cells were adjusted to 1 × 10^6^ cells/ml and then washed two times in PBS with 1% bovine serum albumin (BSA, Sigma– Aldrich) solution. The cell pellets were resuspended and incubated 30 min at 4 °C with the primary antibodies for SLeX, SLeA, LeA and LeX at the same dilutions used for immunofluorescence assay. After two washes, cells were incubated with AlexaFluor647 fluorochrome-conjugate antimouse IgM for SLeX detection and with AlexaFluor488 fluorocrome-conjugate antimouse IgG for SLeA, LeA and LeX, during 30 min at 4 °C. Negative controls were performed by using cells stained only with secondary antibodies. Cells were washed, resuspended in PBS 1% BSA solution and filtered prior analysis. Data acquisition was performed using BD LSRFortessa and analyzed with the FlowJo software (v10; BD Biosciences). Three independent experiments were analyzed.

### SDS-PAGE and western blot analysis

Whole cell lysates were obtained from subconfluent cell cultures using RIPA buffer supplemented with protease and phosphatase inhibitors (Roche). Protein concentration was determined by the bicinchoninic acid protein assay (BCA)(Pierce), and 30 µg of protein extracts were loaded onto 7.5% sodium dodecyl-polyacrylamide gels or 4–15% Mini-PROTEAN® TGX™ Precast Protein Gels, 10 or 15-well (Bio-Rad) for electrophoretic separation (SDS-PAGE) (Bio-Rad). Gels were transferred onto nitrocellulose membranes (Amersham) and blocked with 5% bovine serum albumin (BSA) (Sigma- Aldrich) in PBS containing 0.05% Tween 20 (Sigma-Aldrich) (PBS-T). Western blotting was performed by incubating the membranes with primary antibodies probed overnight (4 °C) followed by incubation with horseradish peroxidase-conjugated goat anti-mouse IgM (1:15000) (Jackson ImmunoResearch) or goat anti-rabbit IgG (1:2000) (Santa Cruz) secondary antibodies. Protein bands were visualized using the ECL WB detection reagent and films (GE Healthcare).

The following primary antibodies were used: Sialyl Lewis X (SLeX, CSLEX, 1:500, BD Bioscience); ß-Actin (1:2000, CellSignaling). ß-Actin was used as loading control.

### Cell proliferation assay

Cell proliferation/viability was analyzed *in vitro* by the tetrazolium salt 3-(4,5-dimethylthiazol-2-yl)-2,5-diphe-nyltetrazolium bromide (MTT) method using a commercially available kit (MTT Cell Proliferation Kit I, Roche). Cells were seeded into 96-well plates (Fisher Scientific) at the density of 1 × 10^4^ cells/well, and MTT Kit solutions added into each well according to cell proliferation kit (Roche) for each time point 24, 48 and 72 h, after seeding. The absorbance value was read at 600 nm using a microplate reader. Three independent assays were performed in triplicates and results are presented as means ± SEM for each sample, and proliferation levels obtained were normalized and compared with the wild-type (WT) cell line.

### Wound healing assay

Cell motility was assessed *in vitro* by a wound healing assay. 2-well silicone ibidi inserts (ibidi) were applied to a 6-well plate (Fisher Scientific) and aproximately 1 × 10^5^ cells were seeded in each side of the 2-well and left to adhere for 24 h. The insert was then carefully removed, leaving a defined gap area (wound) between the cells. After being washed twice with PBS, the cells were then allowed to migrate into the cell-free area. The cells were imaged at 0, 24, 48, and 72 h at a magnification of 100x. The percentage of wound closure was calculated by measuring the free space at each time-point, normalized to the initial wound area (time point-0 h) using ImageJ software (FIJI). Three biological replicates were performed and the results are shown as average ± SEM.

### Statistical analysis

Data were analyzed with GraphPad Prism 8.0 software (GraphPad Software Inc.) Quantitative data were expressed as the mean ± SEM of three biological replicates. To compare the differences between each KO cell model in regard to each respective WT values, a two-way ANOVA with a 95% interval of confidence was performed. P-values of ≤ 0.05 were considered statistically significant.

## Results

### Expression of ST3GalIV and SLeX in gastrointestinal cancer cell lines

**Fig. 1:**
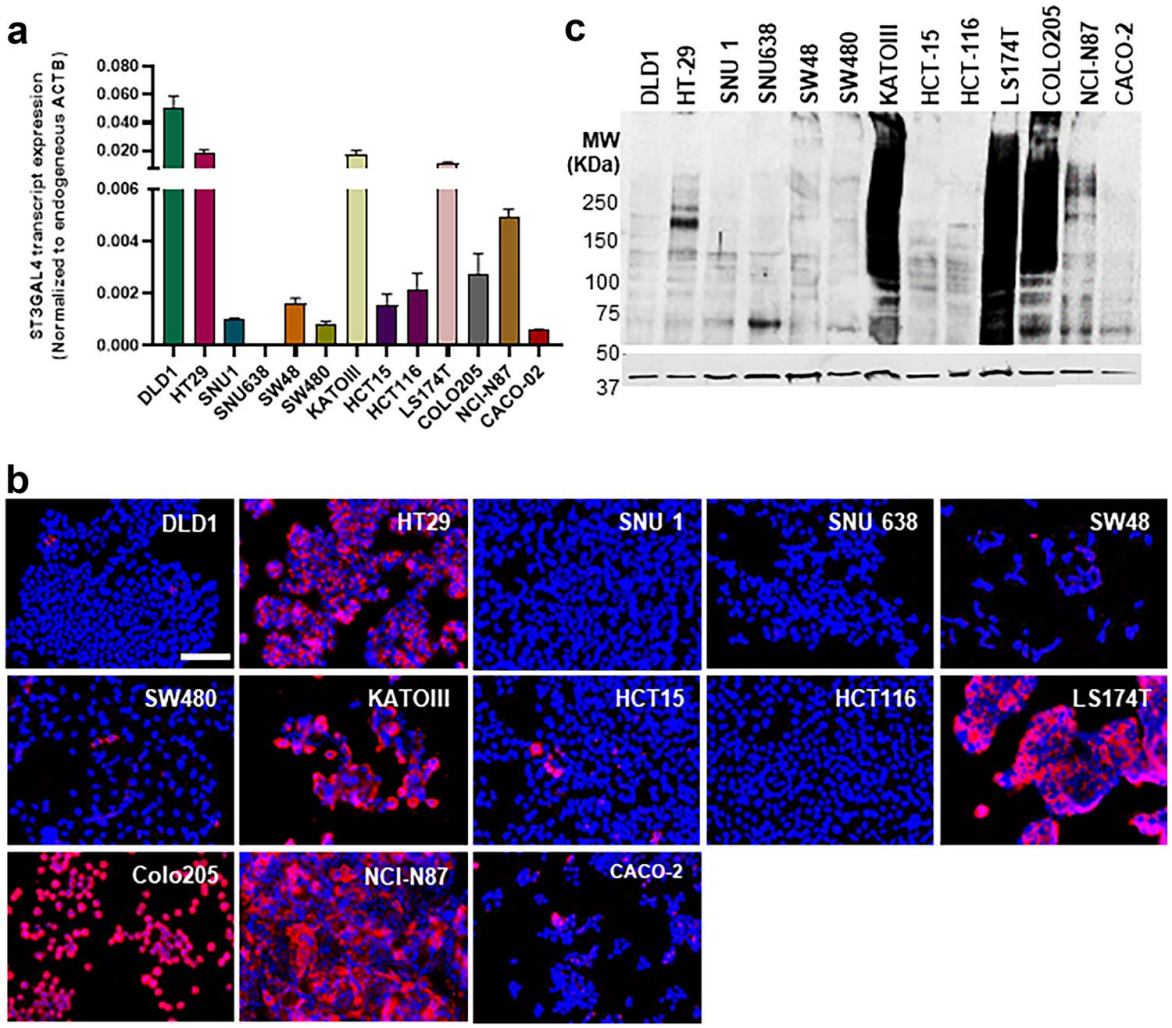
Assessment of ST3GalIV and SLeX expression in a panel of gastrointestinal cancer cell lines. (**a**) ST3GalIV transcript expression levels showing an heterogenous expression across the distinct cancer cell lines, with DLD1, HT-29, KatoIII, LS174T, Colo205 and NCI-N87 presenting the highest levels. Bar graph shows the mean relative quantification values normalized to the internal control gene ACTB. (**b**) Immunofluorescence analysis of SLeX in GI cell lines showing positive staining in HT-29, KatoIII, LS174T, Colo205 and NCI-N87 cell lines. (**c**) SLeX-protein expression by western blot analysis showing a wide range of SLeX positive glycoproteins in HT-29, KatoIII, LS174T, Colo205 and NCI-N87 cells. The scale bar corresponds to 100 μm

To select GI cell lines to further study the role of ST3GalIV in SLeX biosynthesis and expression and malignant characteristics of cancer cells, the transcript levels of ST3GalIV were initially assessed in a panel of 13 GI cancer cell lines. ST3GalIV shows a heterogeneous expression within the studied cell lines (Fig. 1a), with DLD1, HT-29, KatoIII, LS174T, Colo205, and NCI-N87 presenting the highest enzyme levels. Furthermore, we analyzed SLeX expression at the cell surface (Fig. 1b) and specifically at the protein level (Fig. 1c) and identified Kato-III and NCI-N87 (gastric cancer cells) and HT-29, LS174T and Colo205 (colorectal cancer cells) as SLeX positive. Thus, these five SLeX positive cell lines were selected for ST3GalIV gene KO.

### ST3GalIV gene KO impairs SLeX expression mainly at the protein level

**Fig. 2:**
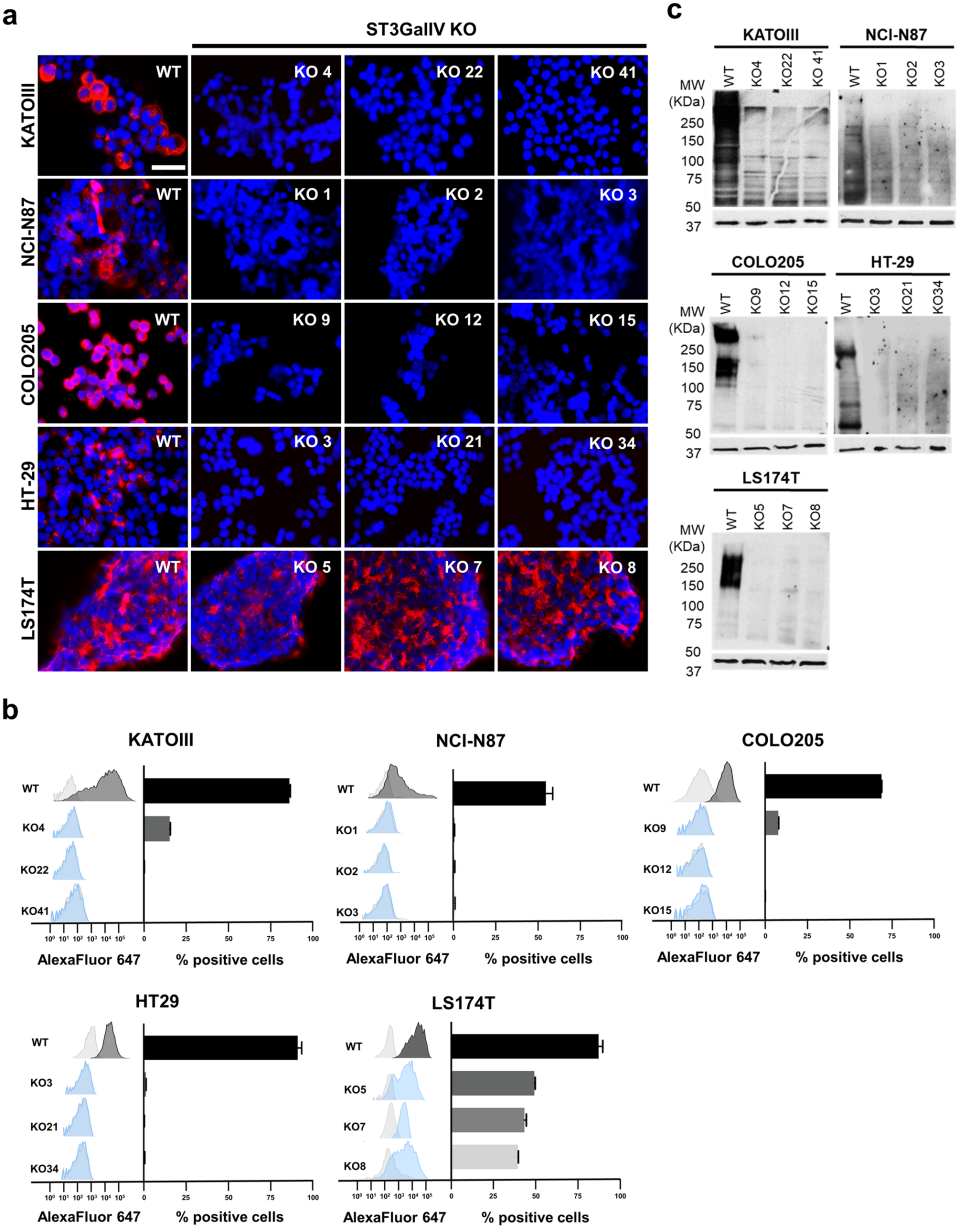
ST3GalIV KO abolished SLeX expression in KatoIII, NCI-N87, Colo205, HT-29 and LS174T cell lines. Immunofluorescence (**a**) and flow cytometry (**b**) analysis showing the abolishment of SLeX in KatoIII, NCI-N87, Colo205 and HT-29 cells upon ST3GalIV KO. The exception is the colon cell line LS174T that still presenting immunodetection at the cell surface. Western blot analysis showed the complete abolishment of SLeX expression upon ST3GalIV KO in all cell lines (**c**). The scale bar corresponds to 50 μm

ST3GalIV KO was performed in the selected cell lines, as previously described using a validated gRNA for *ST3GAL4* [19], and three independent isogenic cell clones were selected for further experiments (Table 2 and Fig. S1). Immunofluorescence analysis for SLeX showed the complete abrogation of SLeX in almost every cell line. The only exception was the colorectal cancer cell line LS174T (Figs. 2a and b and S2). On the other hand, protein analysis by western blot showed the complete abolishment of SLeX expression in all cell lines upon ST3GalIV KO (Fig. 2c). This result pointed to a role of another sialyltransferase in SLeX expression in LS174T cells, likely at the lipid level.

**Table 2:** Summary of ST3GalVI and ST3GalVI indels present in the different gastrointestinal 2 KO cell lines

CELL LINES		ST3GALIV KO	ST3GALIV KO
COLO205	Clone 9	-4/-1	-
	Clone 12	-1/ + 1	-
	Clone 15	-1	-
HT-29	Clone 3	-1	-
	Clone 21	-2/-1	-
	Clone 34	-5/-2	-
LS174T	Clone 5	+ 1	-
	Clone 7	-1/ + 1	-
	Clone 8	-2	-
NCI-N87	Clone 1	-4/-2	-
	Clone 2	-2	-
	Clone 3	-2/-1	-
KATOIII	Clone 4	-2/-1/ + 1	-
	Clone 22	-4/-2/-1	-
	Clone 41	-8/-5/-4/-2/ + 1	-
LS174T	Clone 4	-	-4/-1
	Clone 15	-	-17/-9
	Clone 17	-	+ 1
LS174T	Clone 5	+ 1	-1
	Clone 4	-1/ + 1	-5/-1
	Clone 2	-2	+ 1

**Fig. 3:**
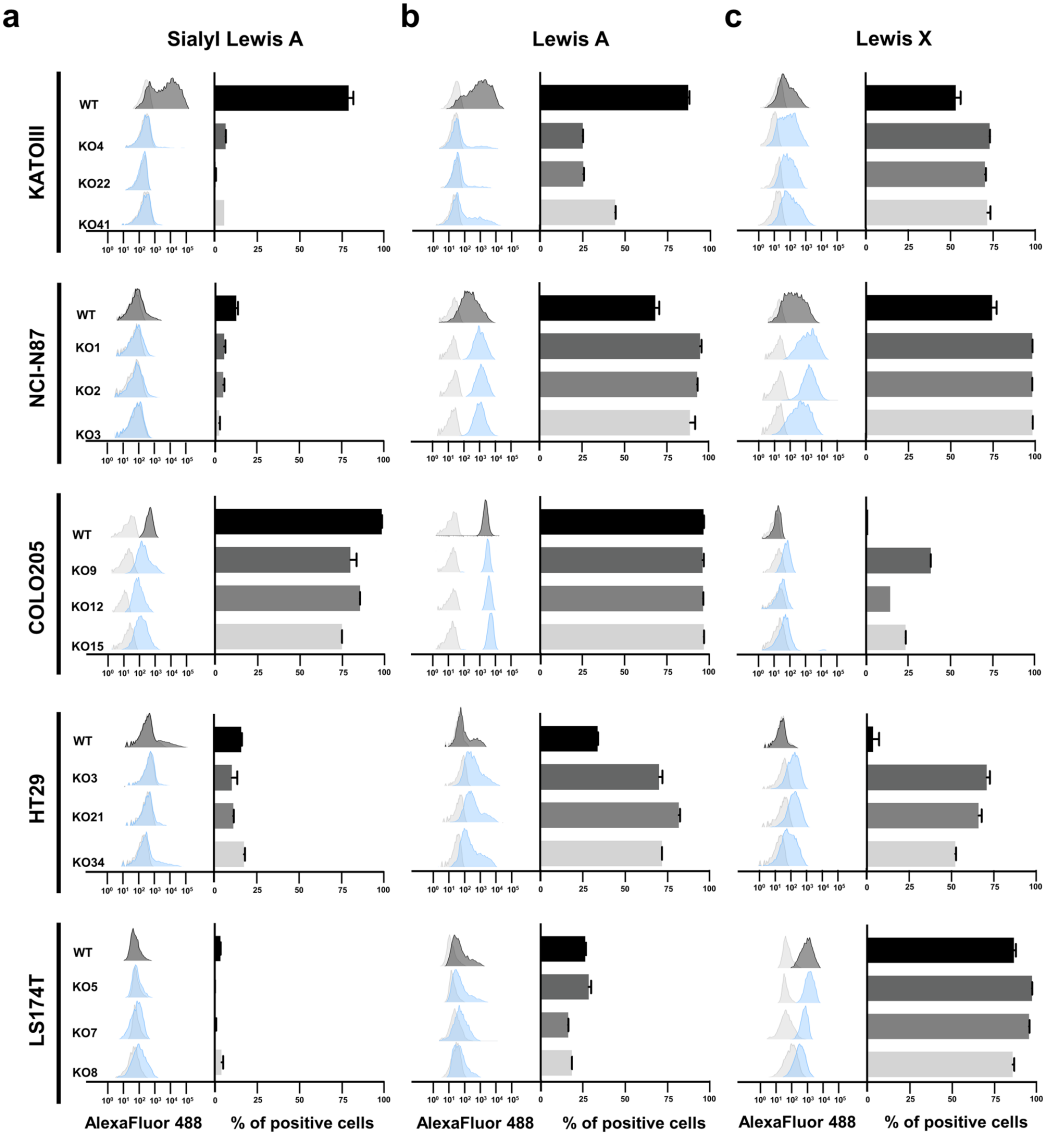
Flow cytometry analysis of SLeA, LeA and LeX in ST3GalIV KO GI cancer cells and parental cells. Levels (histograms) and percentage of positives cells (bar graphs) of cell surface detected SLeA (**a**), LeA (**b**) and LeX (**c**) in KATOIII, NCI-N87, COLO205, HT-29, LS174T. Results showing a heterogenous expression of SLeA, LeA and LeX in the selected WT GI cancer cells. Overall, ST3GalIV KO cells present reduced levels of SLeA detection in positive cells, and an increased detection of the LeA and/or LeX. The only exception is the colon cell line LS174T. Light gray shaded peaks indicate the secondary control

In addition, we evaluated the impact of ST3GAlIV KO in the biosynthesis of the SLeX isomer SLeA and in the non-sialylated Lewis antigens LeA and LeX. Flow cytometry and immunofluorescence analysis in WT cells showed a heterogenous expression of SLeA (Figs. 3 and S3, left panel for each cell line). Colo205 cells presented SLeA positive expression in almost all cells, KatoIII cells presented a high expression with around 80% positive cells and HT-29 cells, NCI-N87 and LS174T cells showed few positive cells (Figs. 3 and S4). Interestingly, ST3GalIV KO led to a reduced SLeA detection in all positive cell lines. Regarding the expression of the non sialylated Lewis antigens LeA and LeX, ST3GalIV KO lead to an increased detection and/or increased number of positive LeA and LeX cells when compared to parental cells (Figs. 3 and S3 middle and right panel for each cell line and Fig. S4). The only exception was the expression of LeA in KatoIII ST3GalIV KO cells that was diminished. Regarding LS174T cells, no no major differences were observed in ST3GalIV KO cells when compared to parental cells for both LeA and LeX detected intensity or number of positive cells (Figs. 3, S3 and S4).

### ST3GalIV-driven SLeX expression impacts the motility of cancer cells

**Fig. 4:**
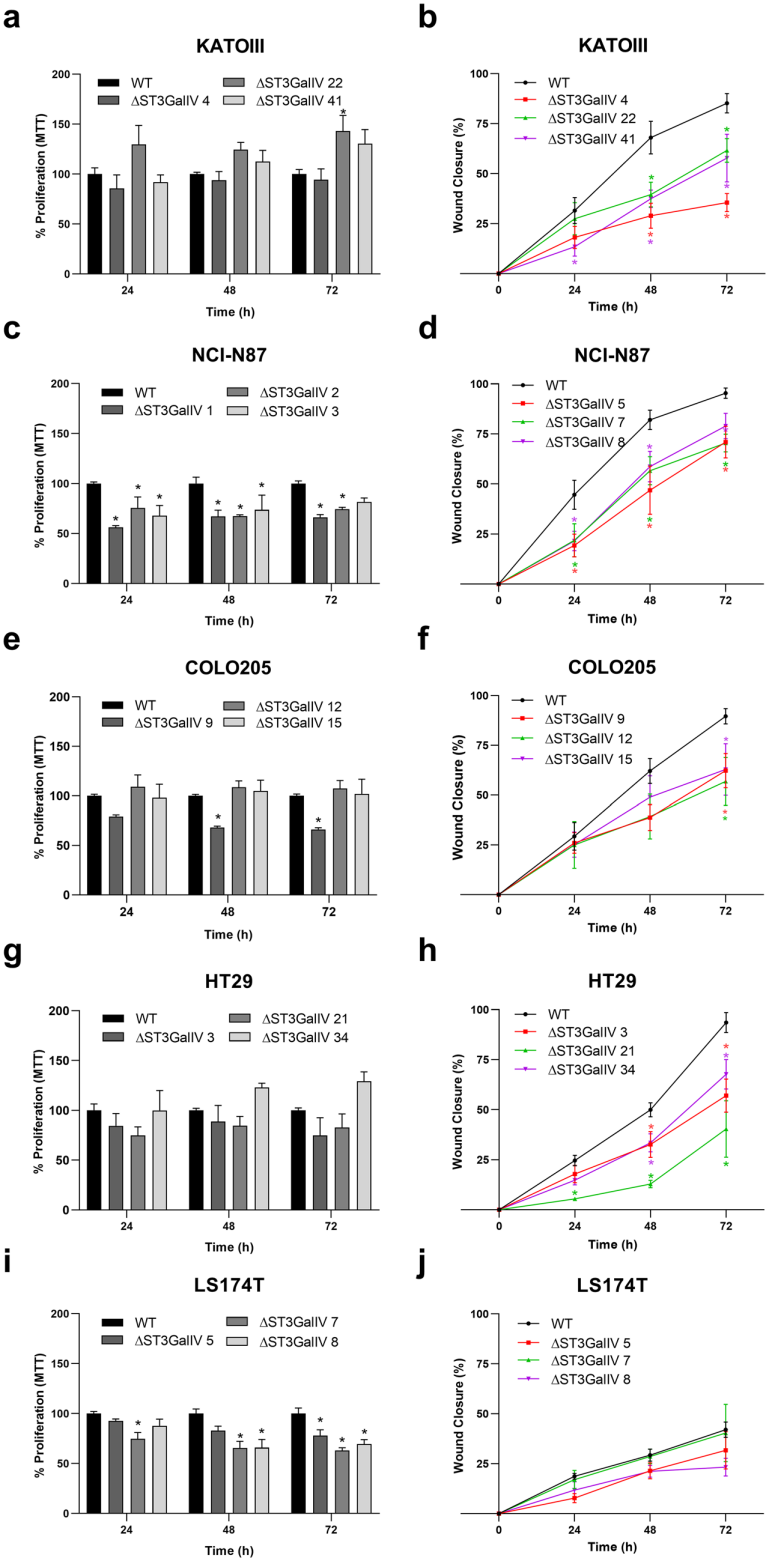
Biological impact of ST3GalIV in the selected GI cancer cell lines. (**a**), (**e**), (**g**), (**i**), showing no differences in cell proliferation upon St3GalIV KO, with the exception of the gastric cancer cells NCI-N87. (**c**), (**b**), (**d**), (**f**), (**h**) Showing the statistically significant reduction in cell motility upon ST3GalIV KO with the exception of LS174T KO cells (**j**). * p ≤ 0.05

To infer the role of ST3GalIV-associated SLeX expression in the malignant properties of cancer cells, we evaluated classical biological parameters such as cell proliferation and cell motility. Results showed that ST3GalIV has a significant impact on the motility capacity of cells, as can be appreciated by the reduced capacity of ST3GalIV KO cells to close the wound along 72 h (Fig. 4b, d, f, and h). The only exception was the LS174T ST3GalIV KO cells, where no differences were observed in cell motility when compared to WT cells (Fig. 4j). Regarding cell proliferation, both NCI-N87 and LS174T ST3GalIV KO cells presented a significant reduction in the proliferation rate assessed at selected time-points, when compared to the parental cell line (Fig. 4c and i). The other KO cell lines presented punctual statistically significant differences (Fig. 4a and e) or no effect (Fig. 4g) when compared to WT parental cells.

### ST3GalVI is also a major contributor for SLeX biosynthesis in LS174T cells and promotes cellular motility

**Fig. 5:**
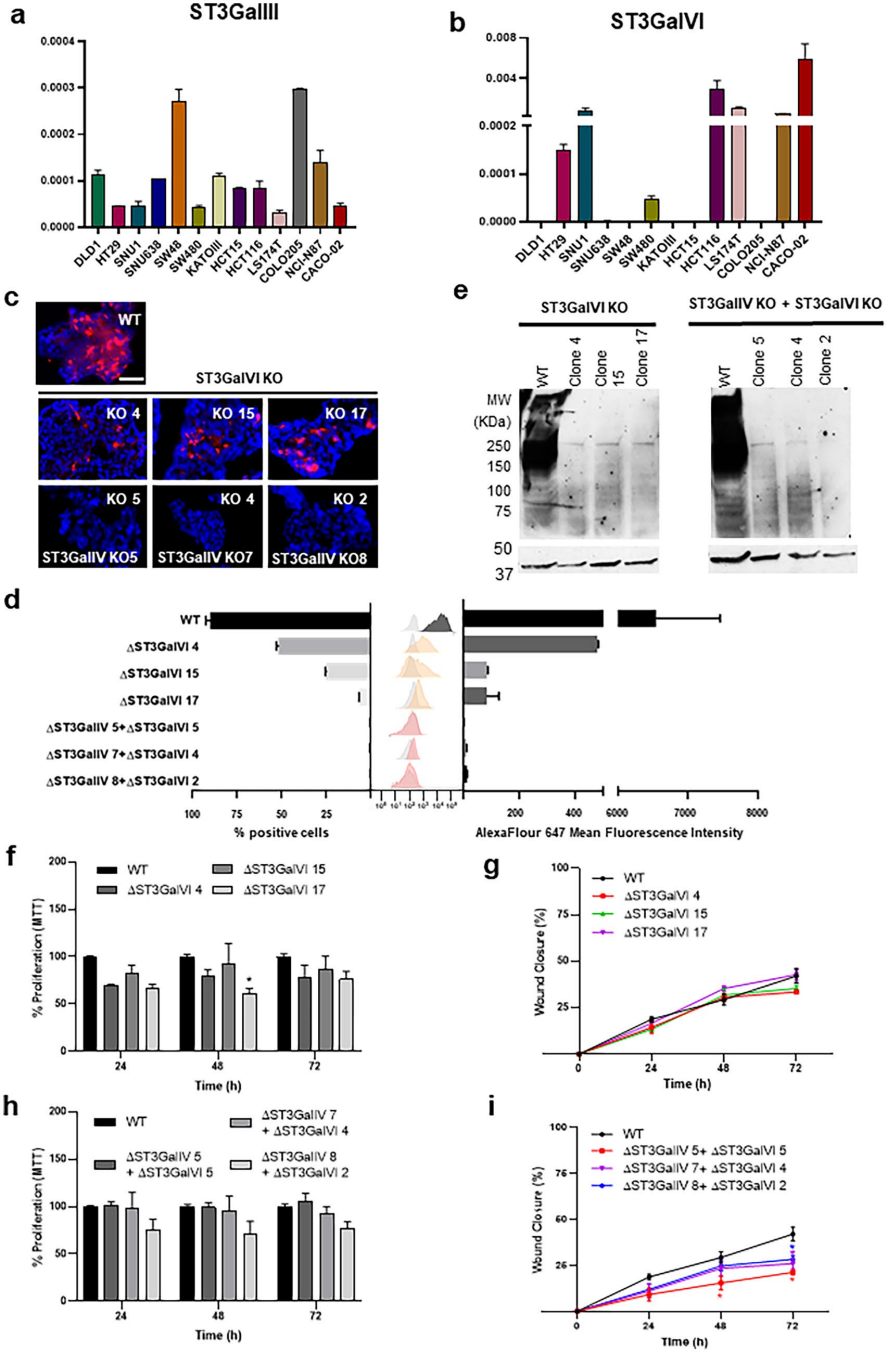
Role of ST3GalVI in SLeX expression and malignant properties of LS174T cells. (**a**) Expression analysis of St3GalIII in a panel of GI cancer cell lines showing a heterogeneous expression within cell lines, with LS174T presenting the lowest expression. (**b**) Expression analysis of St3GalVI in a panel of GI cancer cell lines showing a heterogeneous expression within cell lines, with LS174T presenting a significant expression. Bar graph shows the mean relative quantification values for each gene, normalized to the internal control gene ACTB. (**c**) immunofluorescence analysis of LS174T ST3GalVI KO cells showing a reduced SLeX expression in ST3GalVI KO clones and a complete abolishment of expression in ST3GalIV/VI KO clones. The scale bar corresponds to 50 μm. (**d**) Expression levels and percentage of SLeX positive cells measured by flow cytometry analysis, showing a complete abolishment of SLeX detection in the double KO cells. (**e**) Western blot analysis of ST3GalVI KO clones showing the capacity of ST3GaVI and ST3GalIV/VI KO in impair SLeX expression at the protein level. (**f**), (**h**) Proliferation analysis in ST3GalVI KO clones showing no statistically significant differences. (**g**), (**i**) Motility capacity of ST3GalVI KO clones showing a reduced cell motility only in ST3GalIV/VI KO clones

Since LS174T cells retain SLeX expression after ST3GalIV KO, likely at the lipid level, we sought to investigate the role of other ST3Gals in SLeX biosynthesis in this cell line. We analyzed the expression levels of ST3GalIII (Fig. 5a) and ST3GalVI (Fig. 5b) in the studied cell lines and observed that LS174T expresses both enzymes with significantly levels for ST3GalVI. Since ST3GalVI role in SLeX biosynthesis has been robustly established in previous studies [13, 24, 25], particularly in glycolipids [26], we decided to perform a ST3GalVI KO in LS174T WT cells and in the established LS174T ST3GalVI KO clones. We observed that ST3GalVI KO led to a significant reduction in SLeX in LS174T WT cells (Fig. 5c middle panel and d). In addition, at the protein level we observed that ST3GalVI KO led to the abolishment of SLeX expression (Fig. 5e). The ST3GalVI KO did not have any impact in neither cell proliferation (Fig. 5f) nor motility capacity (Fig. 5g). On the other hand, the ST3GalVI KO in LS174T ST3GalIV KO clones revealed a complete abolishment in SLeX expression (Fig. 5c down panel and d), with a significant impact in the cellular motility (Fig. 5i), but not in the cellular proliferation (Fig. 5h).

## Discussion

SLeX expression is an indisputable feature of immune response activation [27], in addition to playing a pivotal role in leukocyte adhesion and rolling through the endothelium during inflammation [15]. Beyond that, SLeX is increasingly expressed in many carcinomas being associated with motility and invasive properties of cancer cells [8, 11, 18, 28, 29]. Structurally, SLeX is a terminal tetrasaccharide attached to proteins and/or lipids present at the cell surfaces or secreted ones. This tetrasaccharide is composed of a fucose α1,3 linked to N-Acetylglucosamine and a sialic acid α2,3 linked to a galactose, both in type II LacNac chains [30]. The terminal addition of sialic acid in SLeX glycans is described to be performed by members of the ST3Gal family, specifically by ST3GalIII, ST3GalIV, and ST3GalVI. However, it is known that ST3Gal III preferentially acts on type I glycan chains giving rise to the SLeX isomer SLeA, with very low catalytic efficiency towards type II glycan chains [31]. Nevertheless, studies have demonstrated the role of ST3GalIII in SLeX expression for instance, in cancer [32, 33] and in egg implantation [34].

In this study, we wanted to disclose the role of ST3GalIV in SLeX expression and malignant properties of GI cancer cell lines. We prioritized GI cancers as *in vitro* models of disease due to the documented high SLeX expression in these tumors [11]. Additionally, since these cancers are listed on the very top of rankings regarding prevalence and mortality [35], understanding the way SLeX is expressed and impact cancer cell associated biological properties is extremely valuable in the quest for novel therapeutic targets. Thus, we took advantage of an available library of validated gRNAs [19] to specifically abolished the ST3GalIV enzyme expression in SLeX-positive cancer cell lines and observed a complete absence of SLeX expression in the selected clones. In addition, we observed a reduction in SLeA and an overall increased expression of the non sialylated Lewis X and A. The only exception in this study was the LS174T colon cancer cell line, which still retained SLeX expression at the cell surface, only detected by immunofluorescence, suggesting the presence of SLeX-modified glycolipids. In addition, LS174T cells were completely negative for SLeA in both WT and ST3GalIV KO cells. In the late twentieth century, the *in vitro* preference of this enzyme towards type I and II glycan chains, when using ST3GalIV recombinant enzyme, was described and further corroborates our results [36]. Using a soluble form of ST3GalIV from the human placenta, Kitagawa *et al.* also showed the catalytic capacity of this enzyme on both glycoproteins and glycolipids containing type II chains or Galβ1-3GalNAc structures, but not on type I chains [37]. In addition, TNF-induced ST3GalIV expression with consequent SLeX upregulation has been described in lung epithelial cells and human bronchial mucosa [38–40]. Herein, our results, using 4 different ST3GalIV KO GI cancer cell lines, showed the lack of type II sialylation, through the abolishment of SLeX detection, and a reduction in type I sialylation, through the reduction in SLeA detection upon ST3GalIV KO. Thus, our results demonstrate ST3GalIV catalytic capacity on both type I and type II chains. In the literature, it has been shown that ST3GalIII is the major player in SLeA biosynthesis. However, Indellicato and collaborators recently described a nonsense inactivating ST3GAL3 mutation in two patients with congenital disorder of glycosylation in which, contrary to what was expected, it still abundantly expressed circulating SLeA [41]. The authors hypothesized that ST3GAL3 deficiency may partially be compensated by other members of the ST3Gal family and that gangliosides may also play a role. In our work we showed that ST3GalIV KO not only clearly impact the biosynthesis of SLeX but also SLeA. This finding corroborates the above-mentioned study that showed that ST3GAL3 deficiency was not sufficient to impair SLeA biosynthesis, supporting a compensatory coordinated action of STs enzymes.

Further, in colon LS174T cells a likely glycolipid-associated SLeX expression was still observed in ST3GalIV KO cells. Indeed, the major contribution of gangliosides as SLeX/A main carriers has been already characterized by mass spec analysis in WT LS174T cells [42]. Thus, since ST3GalVI was described as acting on type II glycan chains, mostly on glycoproteins and glycolipids [26], we decided to perform ST3GalVI KO in both WT and ST3GalIV KO LS174T. Indeed, ST3GalVI KO on WT cells led to a reduced expression of SLeX expression at the cell surface and its abolishment at the protein level. Furthermore, the double KO led to the complete abrogation of SLeX expression. Nonetheless, the fact that either ST3GalIV or ST3GalVI KO alone led to the complete abolishment of SLeX at the protein level in this cell line is quite puzzling, showing STs redundancy. Regarding this result, we can speculate about an enzymatic complex comprising both enzymes, essential for protein Lewis X sialylation in LS174T cells.

SLeX expression in cancer has been associated with patient’s invasive tumors and dismal prognosis [12, 43], and for this reason, one of our objectives was to evaluate the biological impact of inhibiting SLeX biosynthesis in selected GI cancer cells. We observed that SLeX inhibition did not affect cancer cell proliferation overall. Only NCI-N87 cells showed statistically significant reduction in all the analyzed time-points for the three isogenic clones. The remaining cell lines showed punctual statistically significant differences, that might suggest a clonal selection effect rather than ST3GalIV KO biological effect. However, a complete SLeX inhibition led to a reduced motility capacity in all studied cell lines. This effect was observed in the ST3GalIV KO cells NCI-N87, KatoIII, Colo205 and HT-29 and in the double ST3GalIV/VI KO in LS174T cells. This result corroborates our previous findings that showed an increased invasive capacity of gastric cancer cells when we induced SLeX expression through ST3GalIV overexpression [18]. In addition, other studies that showed the importance of SLeX induction through cytokine stimuli, sowed its role in cancer cell motility and invasion [28].

Overall, our findings demonstrate the role of ST3GalIV in the biosynthesis of SLeX in GI cancer cells, and its association with the cellular motility. Moreover, we showed that, in particular cases (*e.g.* LS174T cells), ST3GalIV is not solely responsible in ensuring SLeX biosynthesis.

## Supplementary Information

Below is the link to the electronic supplementary material.

Supplementary file1 (PDF 15.6 MB)

## Data Availability

The cellular models established during the current study are available from the corresponding author on reasonable request.

## Abbreviations

SLeX: Sialyl Lewis X
SLeA: Sialyl Lewis A
LeA: Lewis A
LeX: Lewis X
α2: β-Galactoside
ST3Gals: 3-Sialyltransferases
CRISPR: Clustered Regularly Interspaced Short Palindromic Repeats
KO: Knock-Out
GI: Gastrointestinal
