# Supplementary material for: ST3GalIV drives SLeX biosynthesis in gastrointestinal cancer cells and associates with cancer cell motility
Source: Glycoconj J. 2023 Apr 19;40(4):421–33. doi: 10.1007/s10719-023-10113-y (PMC10335957; doi:10.1007/s10719-023-10113-y)
Supplement: Supplementary file 1 — Supplementary file1 (PDF 15.6 MB) [file 10719_2023_10113_MOESM1_ESM.pdf]

ST3GalIV KO on COLO205, LS174T, HT-29, NCI-N87 and KatoIII gastrointestinal cancer cell lines

Colo205

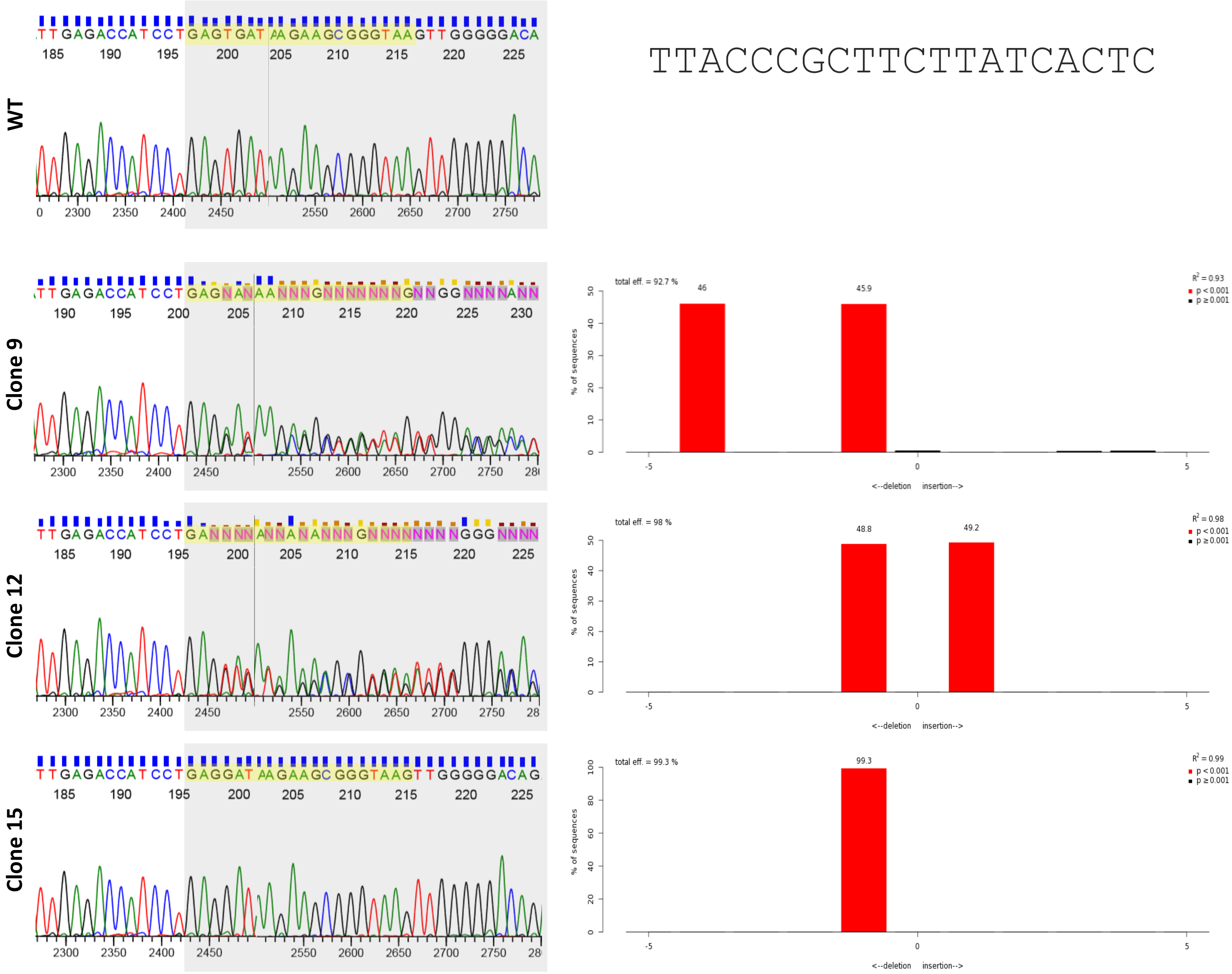

WT
Clone 9
Clone 12
Clone 15
TTACCCGCTTCTTATCACTC
total eff. = 92.7 %
46
45.9
total eff. = 98 %
48.8
49.2
total eff. = 99.3 %
99.3
R^2 = 0.93
R^2 = 0.98
R^2 = 0.99
p < 0.001
p ≥ 0.001
% of sequences
<--deletion insertion-->

NCI-N87

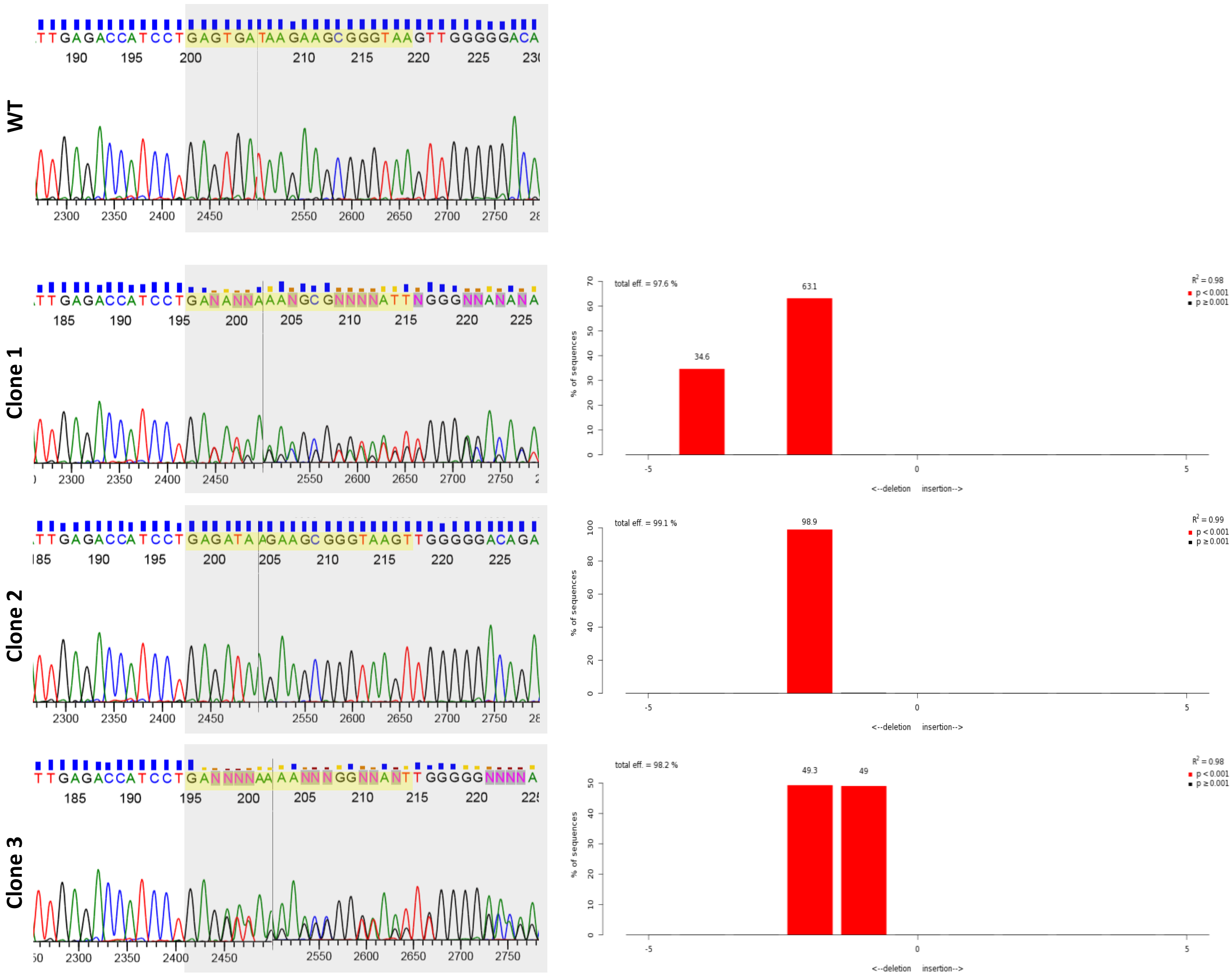

WT
Clone 1
Clone 2
Clone 3
total eff. = 97.6 %
34.6
63.1
total eff. = 99.1 %
98.9
total eff. = 98.2 %
49.3
49
R² = 0.98
R² = 0.99
p < 0.001
p ≥ 0.001
% of sequences
<--deletion insertion-->

HT-29

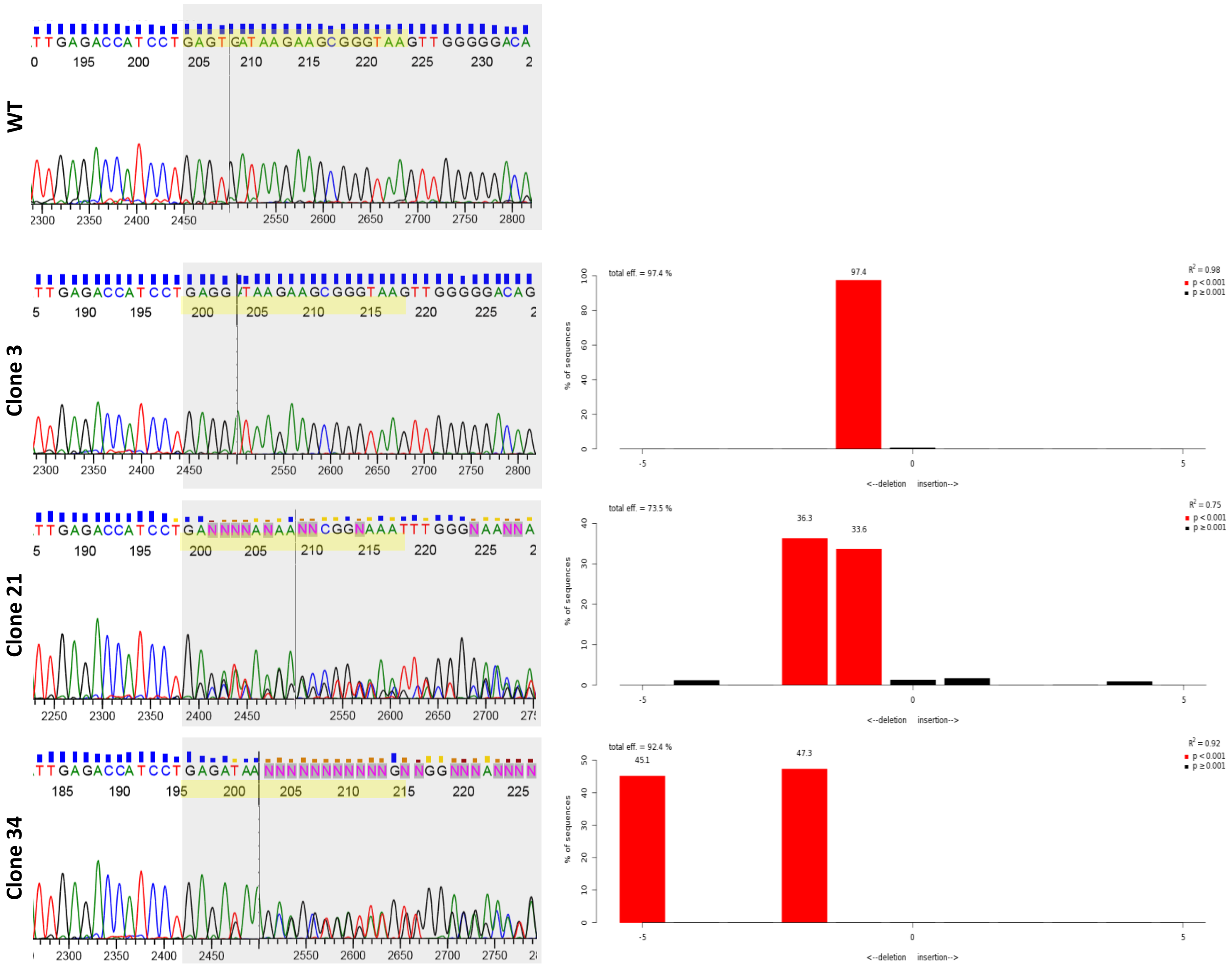

WT
Clone 3
Clone 21
Clone 34
total eff. = 97.4 %
97.4
R² = 0.98
p < 0.001
p ≥ 0.001
total eff. = 73.5 %
36.3
33.6
R² = 0.75
total eff. = 92.4 %
45.1
47.3
R² = 0.92
% of sequences
<--deletion insertion-->

LS174T

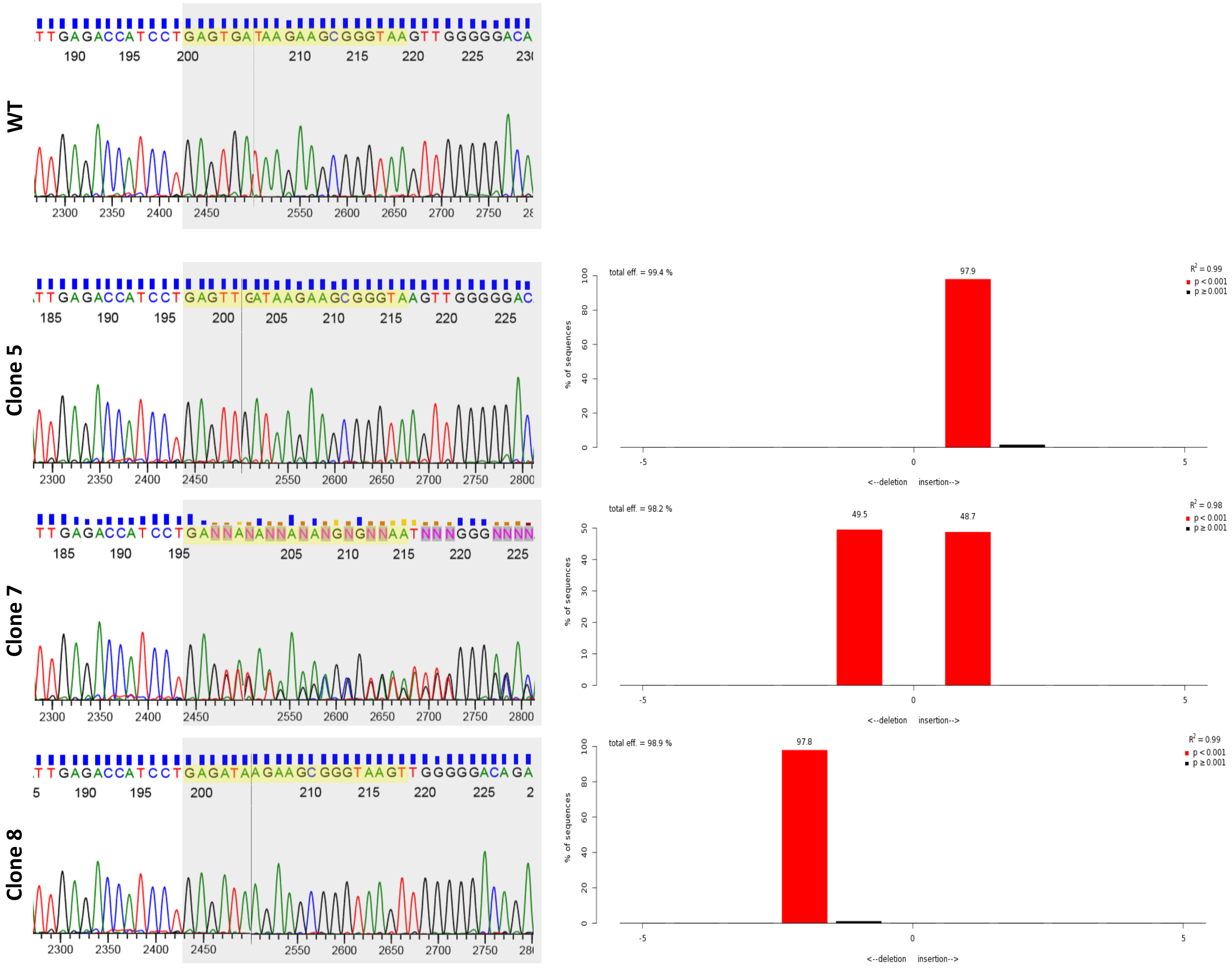

WT
Clone 5
Clone 7
Clone 8
total eff. = 99.4 %
97.9
total eff. = 98.2 %
49.5
48.7
total eff. = 98.9 %
97.8
$R^2 = 0.99$
$R^2 = 0.98$
$p < 0.001$
$p \geq 0.001$
% of sequences
<--deletion insertion-->

Kato III

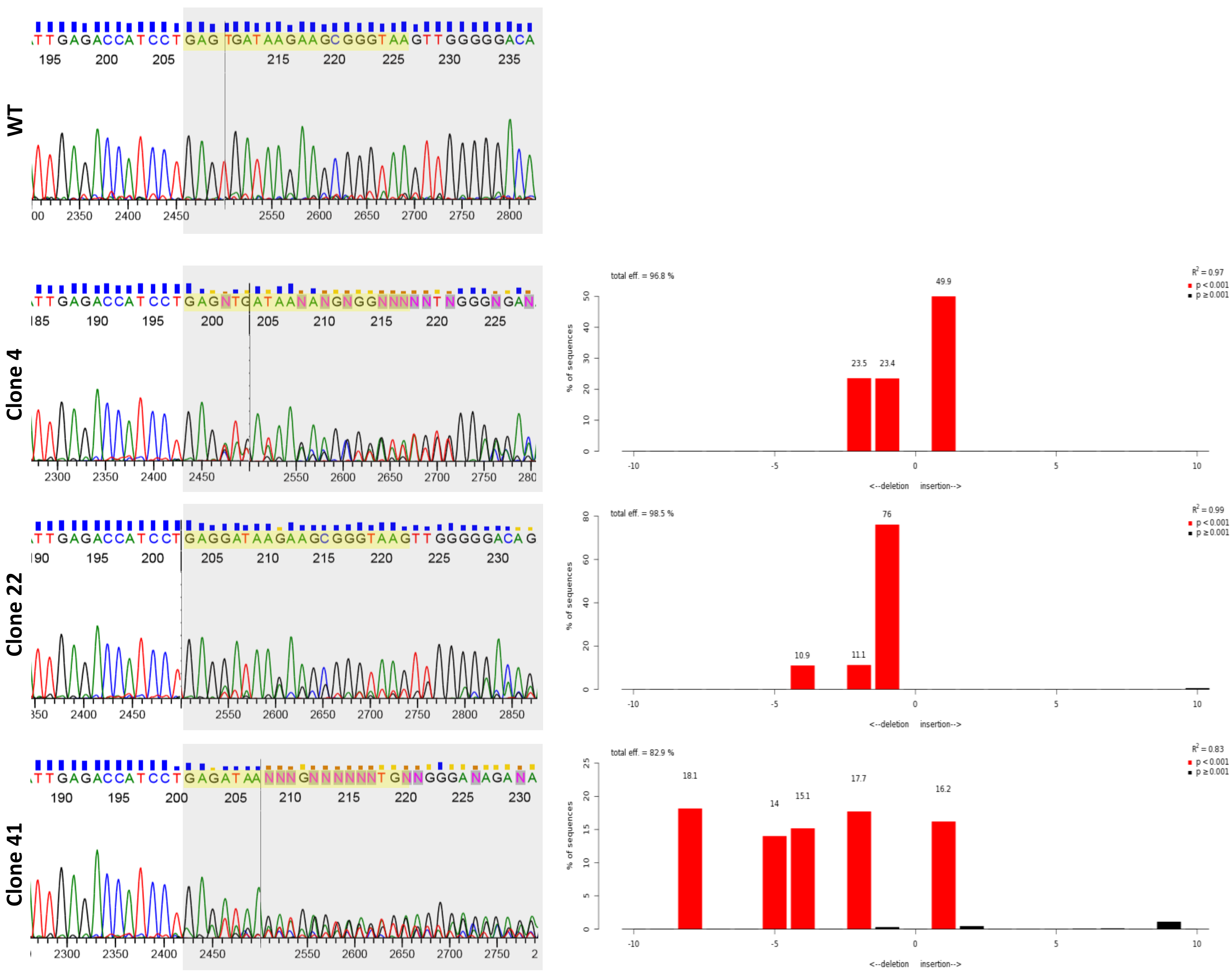
WT
Clone 4
Clone 22
Clone 41
total eff. = 96.8 %
total eff. = 98.5 %
total eff. = 82.9 %
R² = 0.97
R² = 0.99
R² = 0.83
p < 0.001
p ≥ 0.001
% of sequences
<--deletion insertion-->
49.9
23.5
23.4
76
10.9
11.1
18.1
14
15.1
17.7
16.2

ST3GalVI KO on LS174T colon cancer cell lines

LS174T

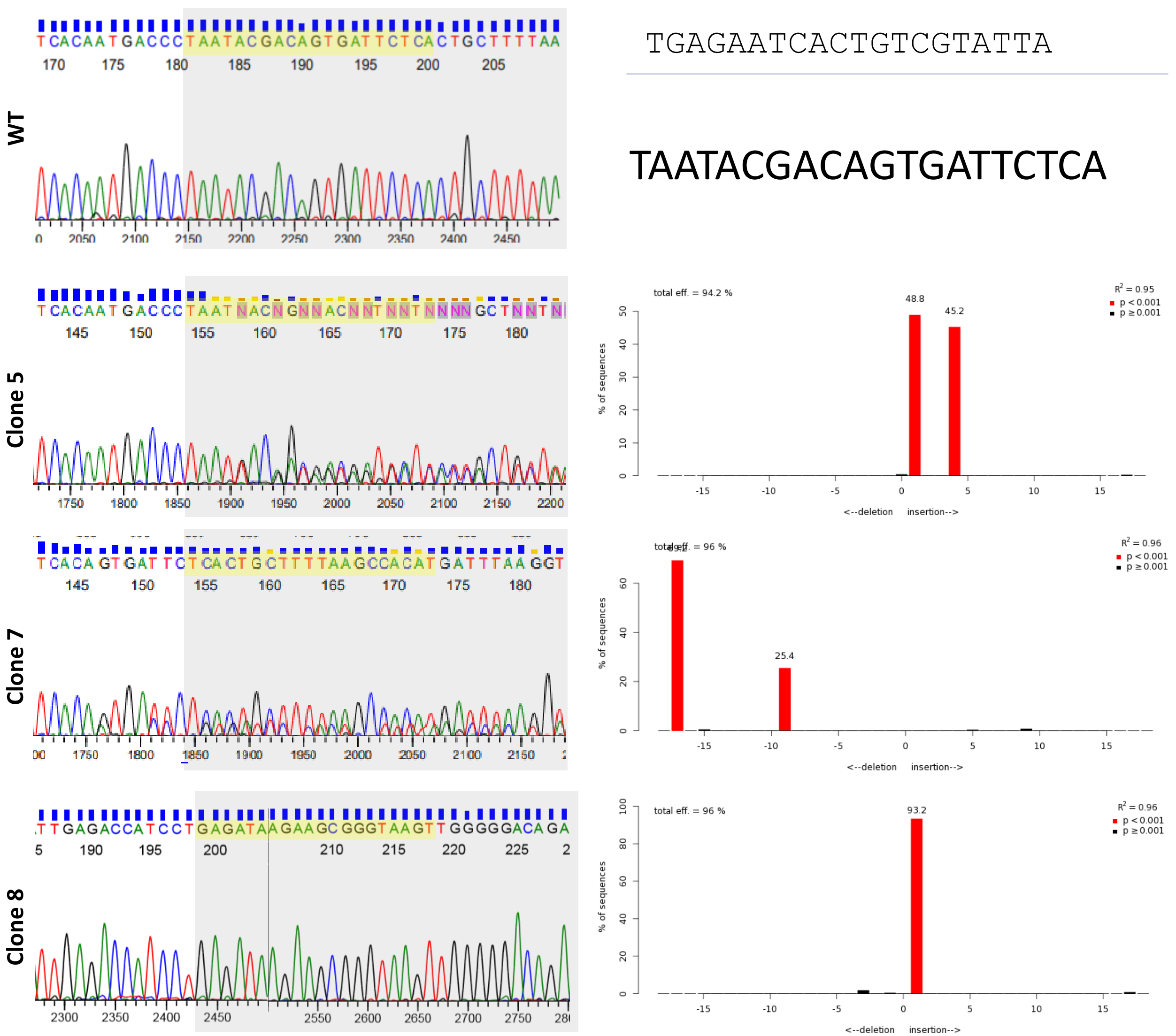

WT
Clone 5
Clone 7
Clone 8
TGAGAATCACTGTCGTATTA
TAATACGACAGTGATTCTCA
total eff. = 94.2 %
R^2 = 0.95
p < 0.001
p ≥ 0.001
48.8
45.2
total eff. = 96 %
R^2 = 0.96
25.4
total eff. = 96 %
R^2 = 0.96
93.2
% of sequences
<--deletion insertion-->

LS174T

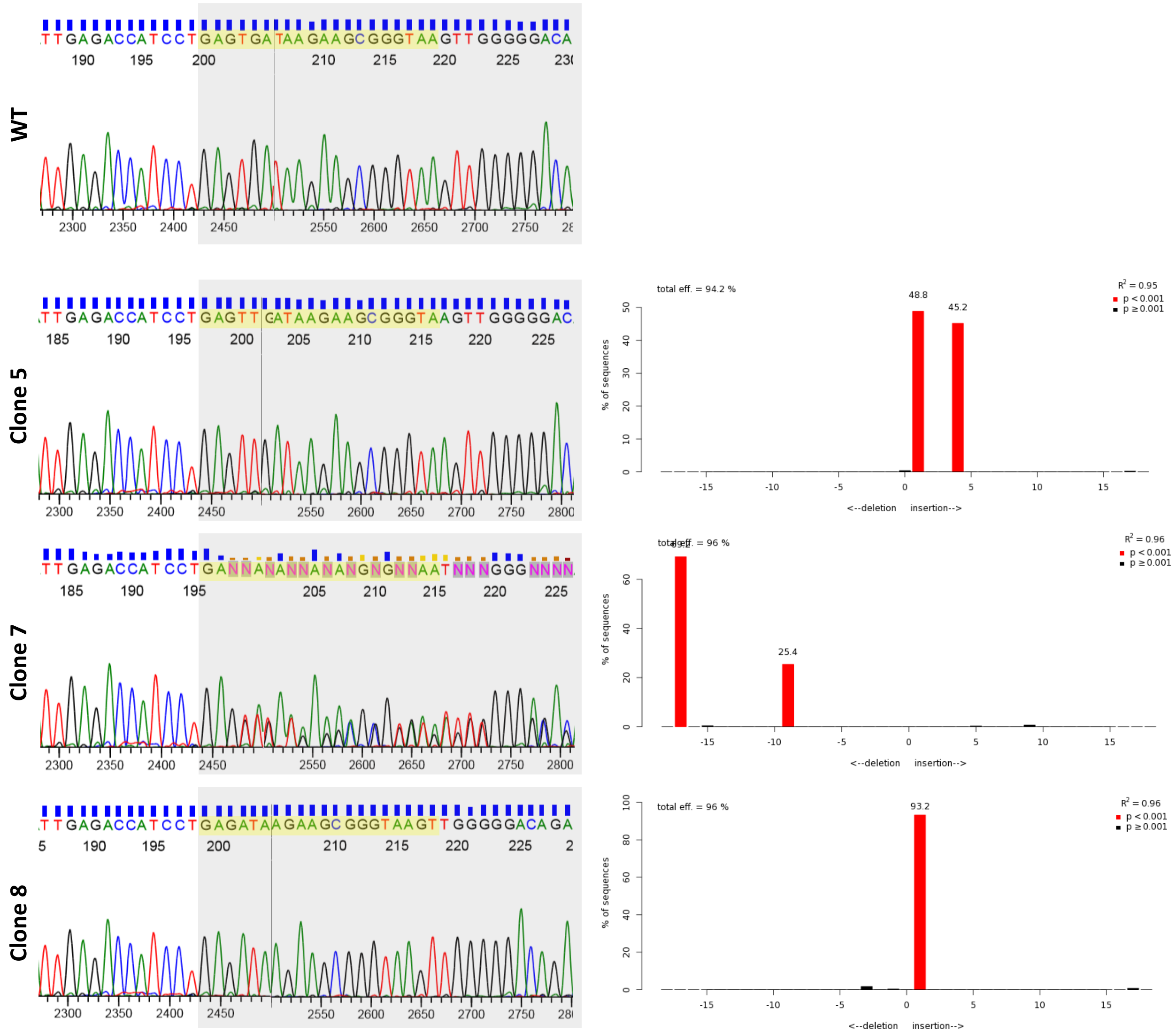
WT
TTGAGACCATCCTGAGTGATAAGAAGCGGGTAAGTTGGGGACA
190 195 200 210 215 220 225 230
2300 2350 2400 2450 2550 2600 2650 2700 2750
Clone 5
TTGAGACCATCCTGAGTTGATAAGAAGCGGGTAAGTTGGGGAC
185 190 195 200 205 210 215 220 225
2300 2350 2400 2450 2550 2600 2650 2700 2750 2800
total eff. = 94.2 %
$R^2 = 0.95$
p < 0.001
p ≥ 0.001
48.8
45.2
% of sequences
<--deletion insertion-->
Clone 7
TTGAGACCATCCTGANNANANNANANGNGNNAATNNNGGGNNNN
185 190 195 205 210 215 220 225
2300 2350 2400 2450 2550 2600 2650 2700 2750 2800
total eff. = 96 %
69.2
$R^2 = 0.96$
p < 0.001
p ≥ 0.001
25.4
% of sequences
<--deletion insertion-->
Clone 8
TTGAGACCATCCTGAGATAAGAAGCGGGTAAGTTGGGGACAGA
190 195 200 210 215 220 225
2300 2350 2400 2450 2550 2600 2650 2700 2750
total eff. = 96 %
$R^2 = 0.96$
p < 0.001
p ≥ 0.001
93.2
% of sequences
<--deletion insertion-->

SFig. 2

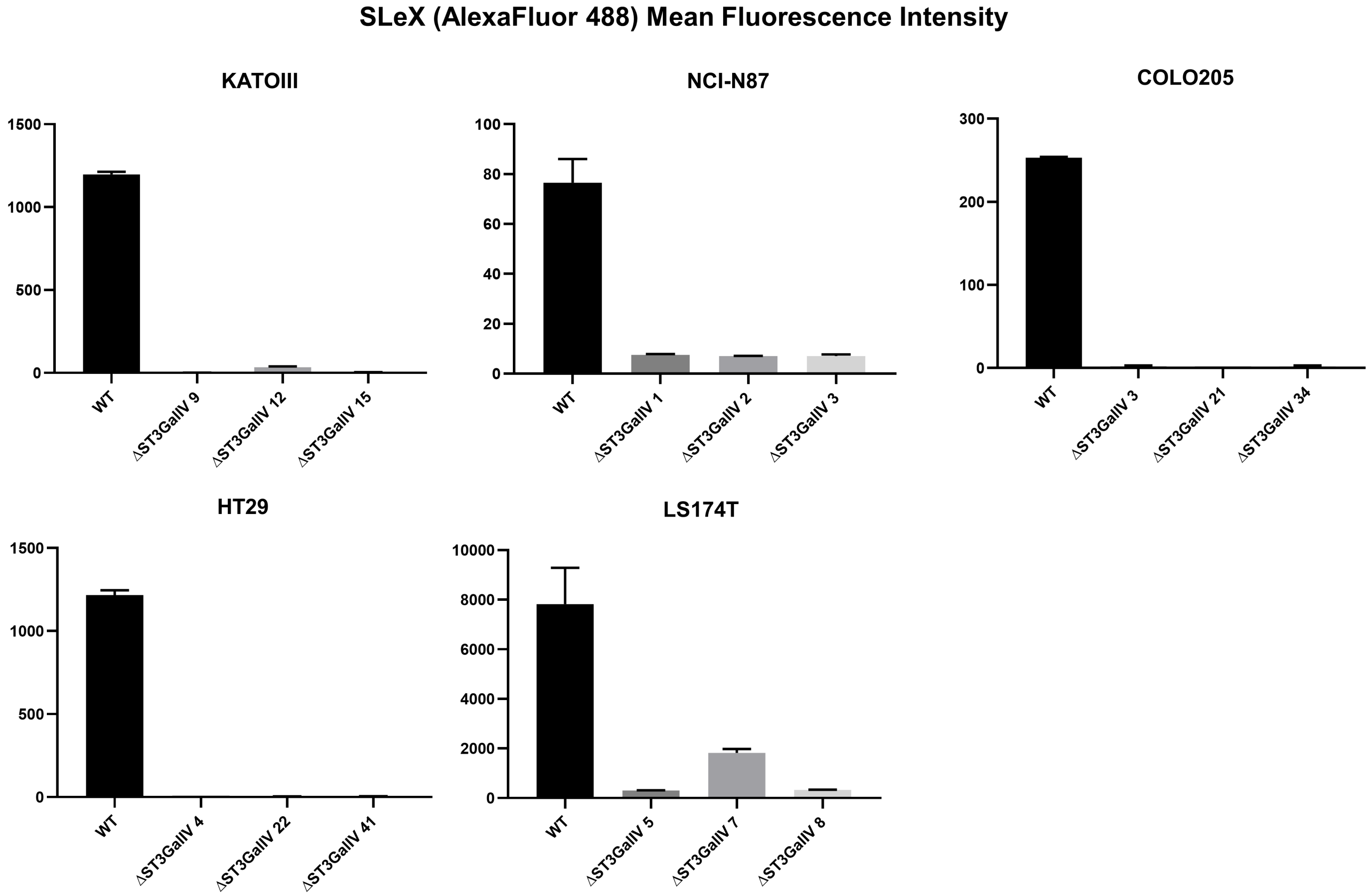

SFig.2: Mean fluorescence intensity levels of cell surface SLeX in WT and ST3GalIV KO clones of KATOIII, NCI-N87, COLO205, HT29 and LS174T cells. Intensity levels were quantified by flow cytometry analysis and shows the full abolishment of SLeX levels in the ST3GalIV KO cells. Bar graphs shows normalized mean intensity of fluorescence (MIF) + SEM, resulting from three independent biological experiments.

SFig. 3

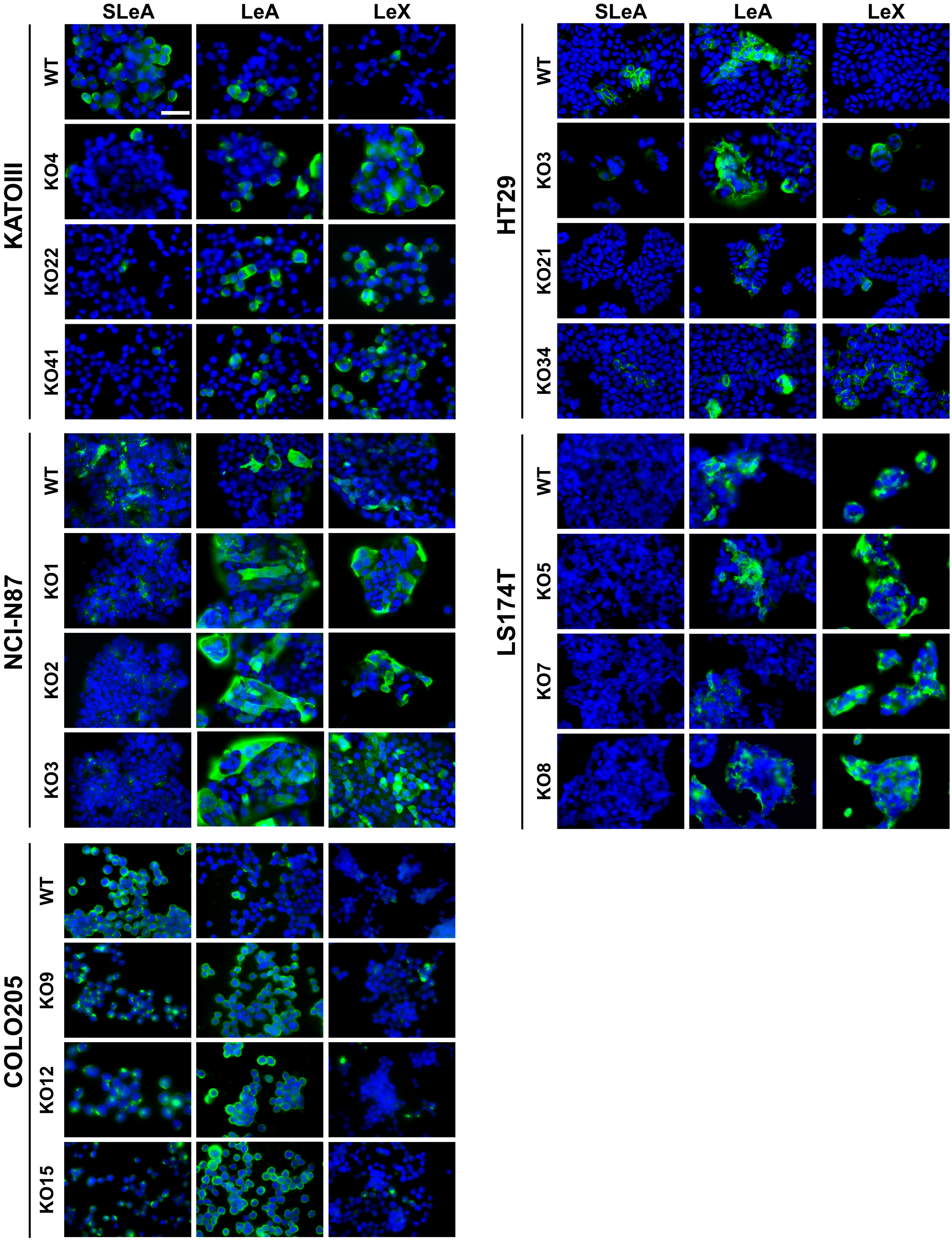

SFig. 3: Immunofluorescence detection of SLeA, LeA and LeX in ST3GalIV KO GI cancer cells. Results showing a heterogenous expression of SLeA in the selected WT GI cancer cells with ST3GalIV KO leading to a reduced expression (left panel for each cell line). ST3GalIV KO also results in the increased expression of the Lewis precursors LeA and LeX (middle and right panel for each cell line). The scale bar corresponds to 50 µm.

SFig 4

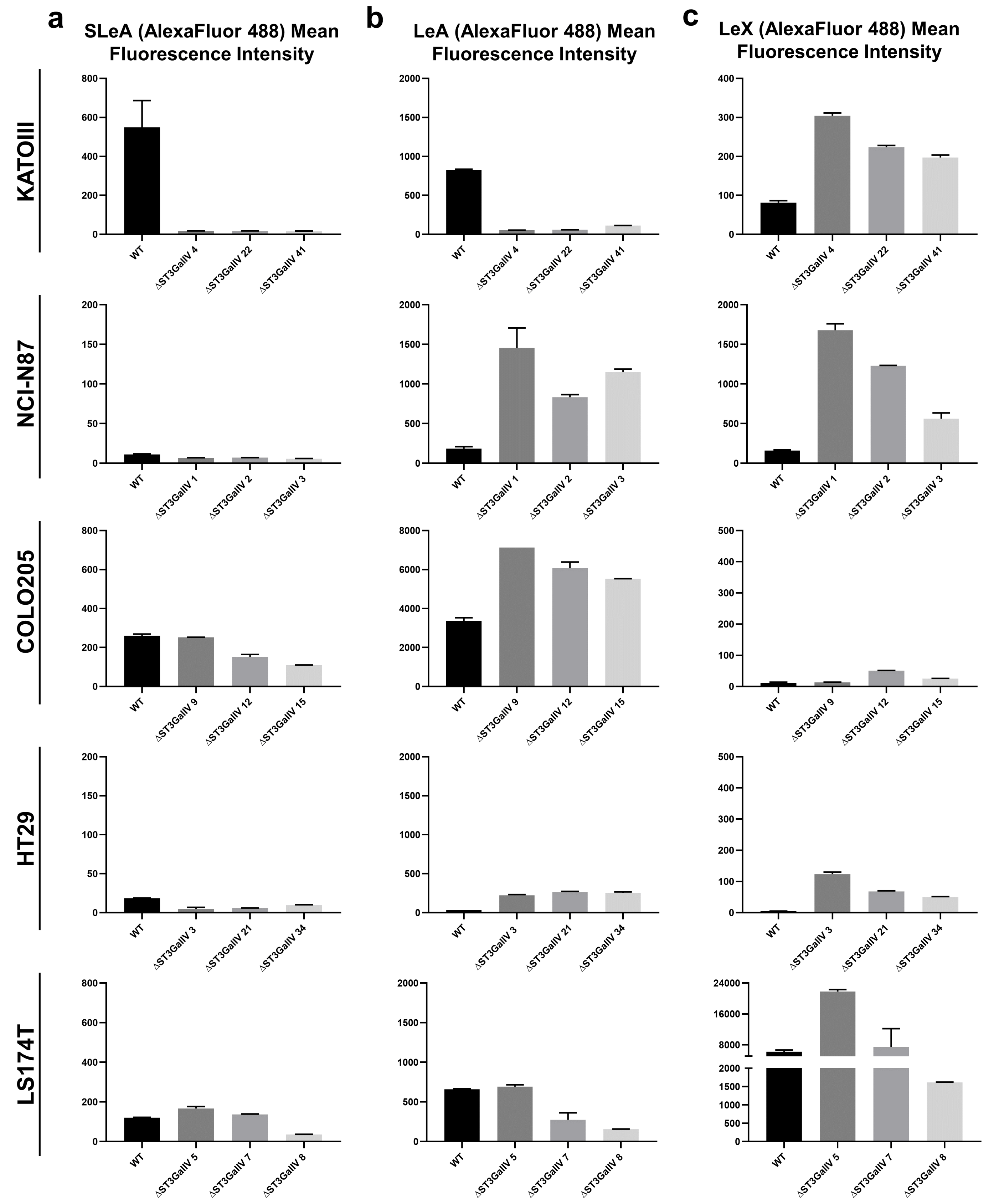

SFig 4: Mean fluorescence intensity levels of cell surface SLeA (a), LeA (b) and LeX (c) in WT and ST3GalIV KO clones of KATOIII, NCI-N87, COLO205, HT29 and LS174T cells. Intensity levels were quantified by flow cytometry analysis. Bar graphs show normalized mean intensity of fluorescence (MIF) + SEM, resulting from three independent biological experiments.
